# Lack of Nck1 protein and Nck-CD3 interaction caused the increment of lipid content in Jurkat T cells

**DOI:** 10.1186/s12860-022-00436-3

**Published:** 2022-07-28

**Authors:** Aussanee Nuiyen, Araya Rattanasri, Piyamaporn Wipa, Sittiruk Roytrakul, Apirath Wangteeraprasert, Sutatip Pongcharoen, Jutaporn Ngoenkam

**Affiliations:** 1 Department of Microbiology and Parasitology, Faculty of Medical Science, Naresuan University, Phitsanulok, 65000 Thailand; 2 Graduate School of Biomedical Sciences Programme, Faculty of Allied Health Sciences, Naresuan University, Phitsanulok, 65000 Thailand; 3 Division of Immunology, Department of Medicine, Faculty of Medicine, Naresuan University, Phitsanulok, 65000 Thailand; 4 Functional Proteomics Technology, National Center for Genetic Engineering and Biotechnology (BIOTECH), Thailand Science Park, Pathumthani, 12120 Thailand; 5 Department of Medicine, Faculty of Medicine, Naresuan University, Phitsanulok, 65000 Thailand

**Keywords:** Nck, Lipid metabolism, Lipid content, Lipid metabolite, Holotomographic microscope

## Abstract

**Background:**

The non-catalytic region of tyrosine kinase (Nck) is an adaptor protein, which is ubiquitously expressed in many types of cells. In T cells, the Nck1 isoform promotes T cell receptor signalling as well as actin polymerisation. However, the role of Nck1 in the lipid metabolism in T cells is unknown. In the present study, we investigated the effect of the Nck1 protein and Nck–CD3 interaction on lipid metabolism and on the physical and biological properties of Jurkat T cells, using a newly developed holotomographic microscope.

**Results:**

Holotomographic microscopy showed that Nck1-knocked-out cells had membrane blebs and were irregular in shape compared to the rounded control cells. The cell size and volume of Nck1-deficient cells were comparable to those of the control cells. Nck1-knocked-out Jurkat T cells had a greater lipid content, lipid mass/cell mass ratio, and lipid metabolite levels than the control cells. Interestingly, treatment with a small molecule, AX-024, which inhibited Nck–CD3 interaction, also caused an increase in the lipid content in wild-type Jurkat T cells, as found in Nck1-deficient cells.

**Conclusions:**

Knockout of Nck1 protein and hindrance of the Nck–CD3 interaction cause the elevation of lipid content in Jurkat T cells.

**Supplementary Information:**

The online version contains supplementary material available at 10.1186/s12860-022-00436-3.

## Background

Human T cells are primarily responsible for the adaptive immune response to invading pathogens and cancer cells [1]. The Jurkat T cell line was derived from the blood of a patient with acute lymphoblastic leukaemia in 1977 [2]. Jurkat T cells are widely used as models to study T cell biology. They express the T-cell antigen receptor (TCR) and CD3 molecules [3]. Triggering of the TCR/CD3 and CD28 pathways induces a complex response of proximal signalling in T cells via CD3ε phosphorylation, followed by downstream signalling [4]. This leads to a change in the T cell metabolism from oxidative phosphorylation to aerobic glycolysis [3]. TCR activation determines cell fate, including cell proliferation, survival, and differentiation, as well as cytokine production [5]. Activation of Jurkat T cells via the CD3ε subunit of CD3 using specific antibodies leads to physiological changes of cell membranes. This activation is also related to F-actin formation, changes in cell shape, and other outcomes [6].

The non-catalytic region of tyrosine kinase (Nck) is an adaptor protein, which plays pivotal roles in many cell types, such as T cells, hepatocytes [7], lens cells [8], and beta cells [9]. In humans, the Nck family has two highly conserved members: Nck1 and Nck2 [10]. Compared to Nck2, Nck1 plays a dominant role in TCR signalling [11]. The Nck protein comprises four domains: N-terminal Src homology (SH) 3.1, SH3.2, SH3.3, and C-terminal SH2. Each domain has several interacting partners [12]. For instance, in T cells, Nck1 interacts with phosphorylated SLP-76 (SH2-domain-containing leucocyte protein of 76 kDa) and recruits several proteins, including Wiskott–Aldrich syndrome protein (WASP), to promote Arp2/3 complex formation that initiates actin polymerisation [10]. Nck1, using its SH3.1 domain, binds the proline-rich sequence (PRS) of CD3ε during T cell activation, leading to intracellular signalling in T cells [13]. Nck1 also interacts with the lymphocyte-specific protein tyrosine kinase (Lck) and T-cell specific adaptor protein (TSAd) to promote TCR signal transduction [14].

The role of Nck in TCR signalling and actin polymerisation has been widely studied [12]. However, there are no reports on the role of Nck in lipid metabolism in Jurkat T cells. Lipid metabolism provides energy to cells and is a precursor for various biological processes. Previously, Lck was reported to play an important role in lipid metabolism by being associated with lipid rafts, which are important for plasma membrane flexibility and TCR clustering [15]. Because Lck is one of the Nck-binding partners [14], we hypothesized that Nck is also involved in lipid metabolism. In the present study, we aimed to assess the role of Nck1 in determining the lipid profile of Jurkat T cells. A newly developed optical diffraction tomography (ODT) or holotomography technique was used to quantify the lipid content with three-dimensional (3D) imaging in label-free samples. In addition, this technique provides information on the physical and biological properties of cells.

## Results

### Nck1 is essential for the maintenance of cell morphology in Jurkat T cells

**Fig. 1:**
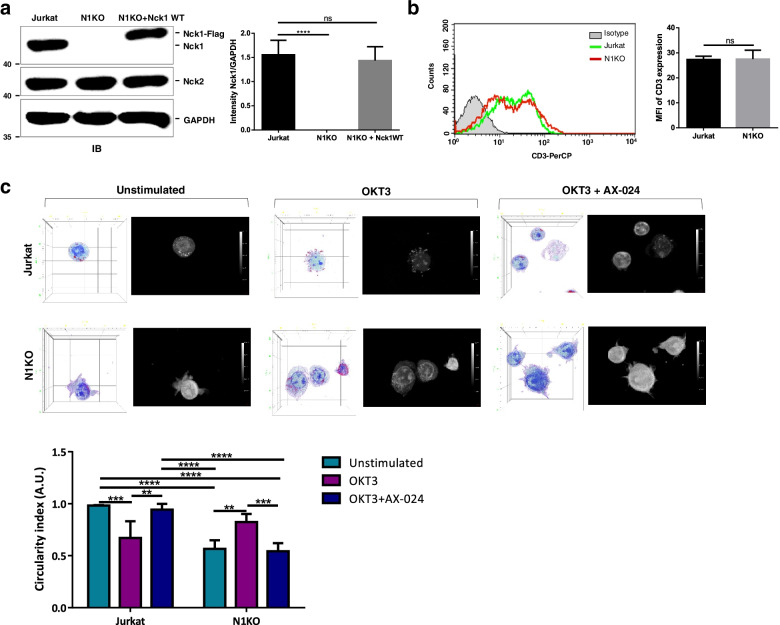
Morphological characteristics of Nck1 knocked-out and Jurkat T cells. **a** CRISPR/Cas9-mediated Nck1 deletion in Jurkat T- cells. Jurkat T cells were transfected with a plasmid containing a guide RNA against *nck1*. A stable clone of Nck1-knockout (N1KO) cells was generated using the limiting dilution technique. Plasmids containing human Flag-tagged wild-type Nck1 were transfected into Nck1-knockout cells. The expression of Nck1 was verified by western blot using antibodies against Nck1, Nck2, and GAPDH (*left panel*). The band intensity of each protein was quantified using ImageJ software and is presented as the ratio of Nck1 to GAPDH. Data are presented as mean ± SD from five independent experiments, *n* = 5 (*right panel*). **b** Nck1-knockout cells (N1KO) had CD3 expression level comparable to that of control Jurkat T-cells. N1KO and Jurkat T cells were stained for surface CD3 molecules with PerCP-conjugated anti-human-CD3ε antibody followed by analysis with flow cytometry. Data are presented as histograms (*left panel*) and bar graphs of mean fluorescence intensity (MFI; *right panel*) from three independent studies (*n* = 3). **c** Deletion of Nck1 causes morphological changes. Cells were left untreated or treated with anti-CD3 antibody (OKT3) alone for 5 min or pre-treated with AX-024 for 30 min, followed by OKT3 stimulation for 5 min. Unlabelled cells were observed under a holotomographic microscope. One representative image from three independent studies of 3D holotomographic (left) and maximum intensity projection (MIP) (right) images is shown, *n* = 3 (*top panel*). Cell circularity was quantified using ImageJ software and presented as the circularity index (A.U) from three separate experiments, *n* = 3 (*button panel*). ns = non-significant; **p < 0.01; ***p < 0.001; ****p < 0.0001

In this study, we used CRISPR/Cas9 to mediate the target gene deletion/knockout of *nck1* in Jurkat T cells (Fig. 1a). Knockout of *nck1* was initially investigated for its effect on surface CD3 expression by flow cytometry. The results showed no significant difference in CD3 expression level between Nck1 knocked-out cells and control Jurkat T cells (Fig. 1b). We then observed the morphology of the Nck1-depleted cells using a holotomographic microscope. In the resting stage, wild-type Jurkat T cells appeared relatively round, whereas knockout of *nck1* in Jurkat T cells resulted in membrane blebs and irregular shapes (Fig. 1c). In the activated stage, wild-type cells had nuclear extension or protrusions, and the cell edge at the cell periphery also had protrusions, causing a drastic morphological change to an irregular shape. In contrast, a relatively round shape was observed in Nck1 knocked-out cells upon stimulation (Fig. 1c).

Since Nck plays a key role in the TCR-proximal and TCR-distal signalling pathways, knockout of the *nck1* was postulated to affect both pathways. To specifically inhibit the function of Nck1 in the TCR-proximal signalling pathway, the small molecule AX-024 was used. [16]. AX-024 was originally reported in 2016 by Borroto et al. to inhibit binding between Nck and CD3ε [16]. AX-024 specifically interacts with the Nck-SH3.1 domain, thereby blocking the association of Nck-SH3.1 domain with CD3ε-PRS upon TCR triggering. Selective inhibition of the Nck–CD3ε interaction by AX-024 causes a defect in actin polymerisation in activated T cells. Furthermore, impaired phosphorylation of CD3ε and ZAP-70 proteins was observed in AX-024-treated cells following TCR-mediated T cell activation [16]. Thus, AX-024 has been found to specifically inhibit TCR-proximal events.

Initially, AX-024 was tested for its effects on cellular toxicity. Compared with untreated cells, AX-024 showed no toxicity to wild-type Jurkat T cells (Supplementary Fig. 1). Interestingly, in the presence of AX-024, Jurkat T cells stimulated with anti-CD3 antibody (OKT3) had clumping nuclei, and the cell shape remained circular, similar to cells in the resting state (Fig. 1c). This appearance was similar to that observed in activated Nck1 knocked-out cells. Stimulation of Nck1 knocked-out cells in the presence of AX-024 caused no alteration of the cell shape since they had an irregular shape, as found in cells in the resting stage. Collectively, these results indicated that a lack of Nck1 and inhibition of the Nck–CD3 interaction affected cell morphology.

### Knockout of Nck1 increased lipid content in Jurkat T cells

The effects of the deficiency of Nck1 on physiological properties in Jurkat T cells were further studied in terms of cell size, cell volume, and lipid content.

**Fig. 2:**
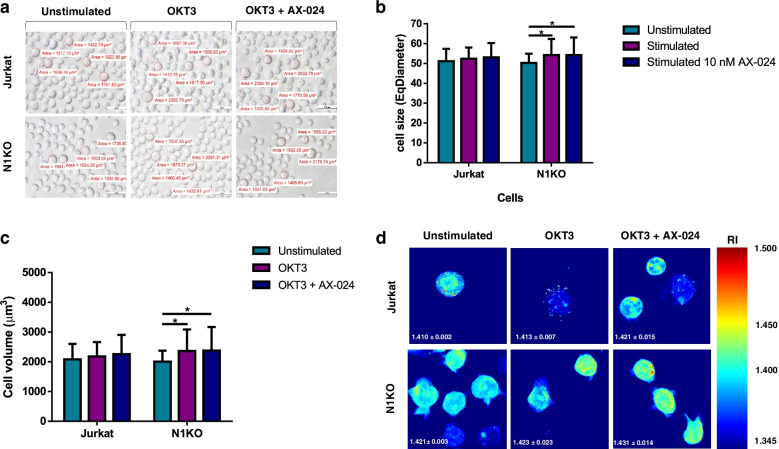
The effects of Nck1 on physiological properties of Jurkat T cell. **a** Cell characteristic under holotomographic microscope. Cells were left untreated or treated with anti-CD3 antibody (OKT3) alone for 5 min or pre-treated with AX-024 for 30 min followed by OKT3 stimulation for 5 min. Un-labelled cells were observed with holotomographic microscope. One representative image from three independent experiments was shown, *n* = 3. **b** cell size, **c** cell volume and **d** refractive index (RI) of wild-type and Nck1 knocked-out Jurkat T cells were identified using Tomostudio software. one representative of topographic images of 3D RI tomograms was shown. The colour bars represented the 3D-rendered RI distribution range (*n* = 1.345 − 1.500). Data are presented as the mean ± SD from three independent studies, *n* = 3.; ns = non-significant; **p < 0.01

The cell size of Nck1 knocked-out Jurkat T cells and control cells was comparable in the resting state (Fig. 2a and b). However, upon stimulation with the OKT3 antibody, the cell size of Nck1 knocked-out Jurkat T cells was significantly increased compared to cells in the resting state, but this was not the case for wild-type cells. Importantly, treatment with AX-024 combined with OKT3 antibody in Nck1 knocked-out Jurkat T cells did not cause any difference in cell size compared to cells stimulated with OKT3 alone. Therefore, upon cell activation, only Nck1 depletion could affect cell size, whereas blocking the Nck–CD3 interaction did not.

In agreement with cell size, the cell volume between Nck1 knocked-out cells and control cells in the resting state was not significantly different (Fig. 2a and c). Interestingly, OKT3-stimulated Nck1 knocked-out Jurkat T cells led to a higher increase in cell volume compared to these cells in resting state. Inhibition of Nck–CD3 interaction using AX-024 did not cause any further change in cell volume in activated Nck1 knocked-out Jurkat T cells compared to cells stimulated with OKT3 alone. Therefore, an increase in cell volume was observed only in Nck1 deficient cells after stimulation.

In addition, the refractive index (RI) distribution of the cells was studied. The RI distribution of the cells was compared with the standard RI values in the holotomographic microscopy database to determine the lipid content in the live cells. Before and after OKT3 stimulation, Nck1 knocked-out Jurkat T cells had a higher lipid content than control Jurkat T cells (Fig. 2d). A significant elevation in lipid content in wild-type cells was observed when these cells were activated in the presence of AX-024. Treatment with AX-024 enhanced lipid accumulation in Nck1-knocked-out cells upon stimulation. Thus, deficiency of the Nck1 protein and blocking of Nck–CD3 binding caused an elevated lipid content within the cells.

### Upregulation of lipid metabolites in Nck1 knocked-out Jurkat T cells

**Fig. 3:**
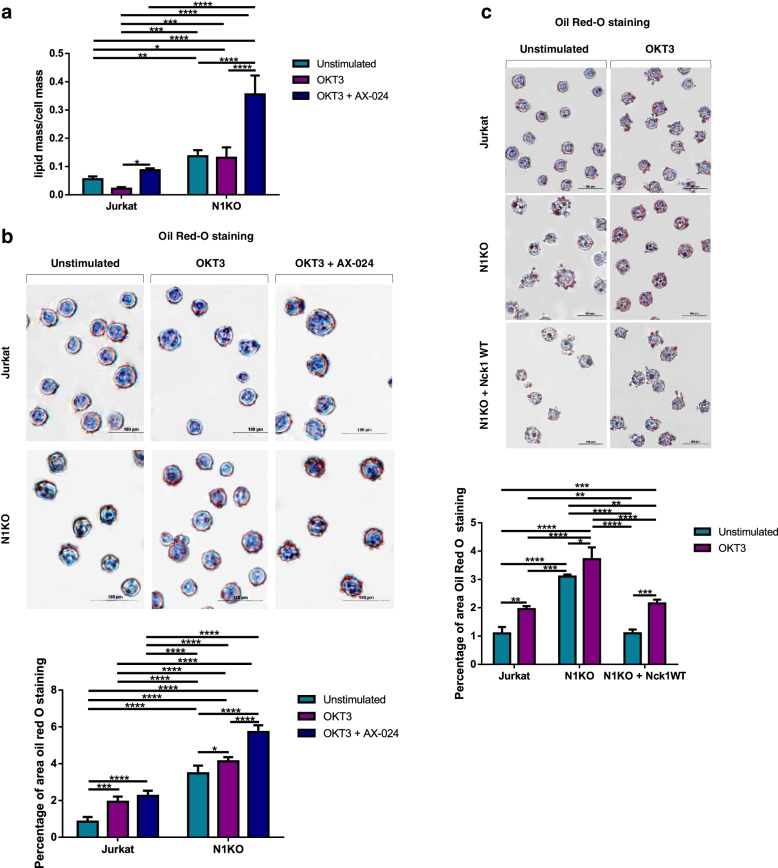
The effects of Nck1 on lipid content in Jurkat T cell. **a** Deficiency of Nck1 and inhibition of Nck–CD3 interaction caused an increase of lipid mass/cell mass ratio. Cells were unstimulated or stimulated with anti-CD3 antibody (OKT3) alone for 5 min or pre-treated with AX-024 for 30 min followed by OKT3 stimulation for 5 min. Lipid mass/cell mass ratio was monitored by holotomographic microscopy and analysed by Tomostudio software. Data are represented as the mean ± SD from three independent studies, *n* = 3. **b** Deletion of Nck1 and inhibition of Nck/CD3 binding was associated with increase lipid droplet. Cells were treated as above and stained with Oil Red O before observing under the inverted microscopy. Image represented one from five separated experiments, *n* = 5 (*top panel*). Lipid content in cells was quantified using ImageJ software and presented as mean ± SD of the percentage of Oil Red O-positive area from three independent experiments (*button panel*). **c** Re-constituted Nck1-depleted cells (N1KO) with wild-type Nck1 reverted the amount of lipid droplet to normal as Jurkat T cells. N1KO cells were re-constituted with wild-type Nck1 and treated as above before conducting the Oil Red O staining. Picture represented one from three separated experiments, *n* = 3 (*top panel*). Lipid droplet in cells was quantified by ImageJ software and presented as mean ± SD of the percentage of Oil Red O-positive area from three independent experiments, *n* = 3 (*button panel*). ns = non-significant, **p < 0.01, ***p < 0.001, ****p < 0.0001

Alteration of RI distribution in Nck1 knocked-out Jurkat T cells indicated that Nck1 is involved in determining lipid content. Thus, the lipid mass/cell mass ratio was determined using a holotomography microscope, which has been used in quantitative image analysis of lipid content in living cells without labelling agents [17, 18]. Nck1 knocked-out Jurkat T cells had a higher lipid mass/cell mass ratio than control Jurkat T cells, irrespective of stimulation (Fig. 3a). These results suggested that Nck1 is involved in lipid accumulation in T cells. To prove this, lipid droplets in Nck1 deficient cells were determined using the Oil Red O technique (Fig. 3b). A marked increase in lipid droplets was observed in Nck1 knocked-out Jurkat T cells before and after stimulation, suggesting the involvement of Nck1 in lipid metabolism. Importantly, in the presence of AX-024, the amount of lipid droplets increased significantly in OKT3-stimulated wild-type cells Nck1 knocked-out cells compared to cells treated with OKT3 alone. To further verify that the increase in lipid droplets was caused by Nck1 protein depletion, Nck1 knocked-out cells were re-constituted with the wild-type *nck1* (Fig. 1a); subsequently the lipid droplets were determined using the Oil Red-O technique. The quantity of lipid droplets in the re-constituted Jurkat cells was equivalent to that in wild-type Jurkat T cells (Fig. 3c). Altogether, Nck1 deficiency and inhibition of the Nck–CD3ε association were related to increased lipid droplets in Jurkat T cells.

**Fig. 4:**
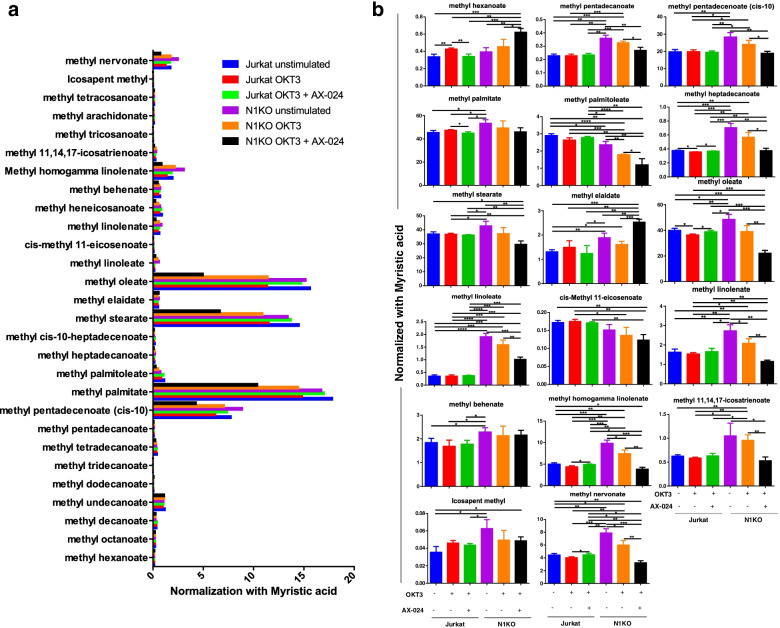
The effects of Nck1 deficiency on lipid metabolite profile in Jurkat T cell. **a** Lipid metabolite profiles of Nck1-deficient cells. Cells were unstimulated or stimulated with anti-CD3 antibody (OKT3) alone for 5 min or pre-treated with AX-024 for 30 min followed by OKT3 stimulation for 5 min. Cells were processed for lipid metabolites analysis using Gas-Chromatography-Quadrupole Time of Flight mass spectrometer (GC-QTOF). Level of each of lipid metabolites was normalized by myristic acid and presented as bar graph. One representative image from three independent studies was depicted, *n* = 3. **b** Nck1 knock-out caused an increase of various lipid metabolites. Cells were treated and analysed as above. Selected of lipid metabolites were shown. Data were representative of three separated experiments, *n* = 3 (mean ± SD). ns = non-significant, *p < 0.05, **p < 0.01, ***p < 0.001, ****p < 0.0001

These results indicate that Nck1 is involved in lipid content regulation. Lipid metabolites in Nck1 knocked-out cells were further assessed using gas chromatography-quadrupole time-of-flight mass spectrometry (GC-QTOF). In the present study, twenty-eight lipid metabolites were detected in Jurkat T-cells (Fig. 4a). Among these, levels of 17 lipid metabolites were significantly different compared to those in control cells (Fig. 4b). Compared to the control cells in the resting state, resting Nck1 knocked-out Jurkat T cells significantly upregulated 13 lipid metabolites, including methyl pentadecanoate, methyl pentadecenoate (cis-10), methyl palmitate, methyl heptadecanoate, methyl stearate, methyl elaidate, methyl oleate, methyl linoleate, methyl linolenate, methyl behenate, methyl homogamma linolenate, icosapent methyl, and methyl nervonate (Fig. 4b). Compared to the resting state, these lipid metabolites were slightly decreased upon activation of Nck1 knocked-out Jurkat T cells, with a significant decrease observed for methyl linolenate, methyl homogamma linolenate, and methyl nervonate. In contrast to those in Nck1-depleted cells, the levels of most lipid metabolites were comparable between resting and activated wild-type Jurkat T cells (Fig. 4b). Compared to resting wild-type Jurkat T cells, cis-methyl 11-eicosenoate slightly decreased, and methyl palmitoleate was markedly decreased in resting Nck1 deficiency cells (Fig. 4b). Interestingly, treatment with AX-024 followed by OKT3 stimulation in Nck1 knocked-out Jurkat T cells was associated with a reduction in all lipid metabolites compared to cells stimulated with OKT3 alone, except for methyl hexanoate and methyl elaidate. The increased levels of lipid metabolites in Nck1-depleted cells correlated with the results of lipid content, lipid mass/cell mass, and lipid droplets. These results suggest that Nck1 deficiency increased the lipid content in resting Jurkat T cells.

### Nck1 deficiency affected actin polymerization in Jurkat T cells

**Fig. 5:**
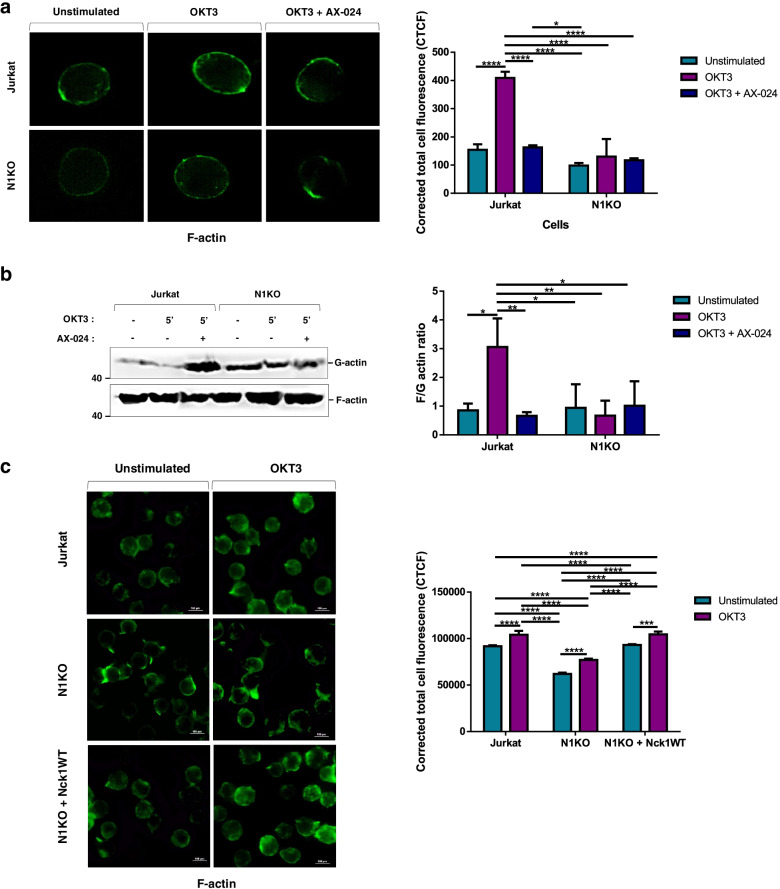
Nck1 depletion and Nck–CD3 interaction inhibition affected on actin polymerization in Jurkat T cells. **a** Deletion of Nck1 and inhibition of Nck–CD3 association impaired actin polymerization. Cells were untreated or stimulated with 5 μg/mL of anti-CD3ε antibody (OKT3) in the presence or absence of 10 nM AX-024. Cells were stained for fibrous-actin filament with rhodamine-phalloidin-conjugated FITC (green) then observed under holotomographic microscope (Tomocube). One representative picture of each sample was cropped for presentation purpose and three independent experiments were done, *n* = 3, (*left panel*). ImageJ software was used to measure the value of integrated density, area of selected cell, and mean fluorescence of background before calculating the Corrected total fluorescence (CTCF) intensity. Data represented the mean ±  SD of CTCF intensity from three separated experiments, *n* = 3 (*right panel*). **b** Decreased F-actin/G-actin ratio was found in Nck1-depleted cells and Nck–CD3 interaction inhibition. Cells were treated as above and fractions of G-actin and F-actin were prepared before subjecting to immunoblot analysis using antibody against actin. One representative immunoblot of three independent studies was shown (*left panel*). Band intensity of G-actin and F-actin were quantified using ImageJ software, and the ratio of F-actin/G-action was then calculated. Data are represented as the mean ± SD of band intensity from three independent experiments, *n* = 3 (right panel). **c** Re-expression of wild-type Nck1 in Nck1 knocked-out (N1KO) cells rescued actin polymerization. Cells were treated and stained with rhodamine-phalloidin-conjugated FITC (green) as indicated above followed by observing under the fluorescent microscope. One representative fluorescent image of three independent experiments were shown, *n* = 3, (*left panel*). ImageJ software was used to analyse for the CTCF intensity. Data are represented as the mean ± SD of CTCF intensity from three separated experiments, *n* = 3 (*right panel*). ns = non-significant, *p < 0.05, **p < 0.01, ***p < 0.001, ****p < 0.0001

In terms of physiological properties, Nck1-knocked-out Jurkat T cells showed alterations in cellular shape when compared with control cells. Nck1 plays a key role in regulating actin filament formation, providing mechanical support, and determining cell shape [14]. Therefore, the alteration of cell morphology in Nck1-depleted cells might be due to a defect in actin polymerisation. To confirm this, cells were stained with phalloidin-conjugated FITC to observe fibrous actin network formation (F-actin) under a holotomographic microscope. The results showed that OKT3-stimulated wild-type cells had a complete circular ring of F-actin at the periphery (Fig. 5a). In contrast, Nck1 depleted T cells showed an incomplete circular ring at the cell edge, indicating impaired actin polymerisation. Interestingly, wild-type Jurkat T cells treated with AX-024 and stimulated with OKT3 were also associated with an impairment of actin polymerisation, as found in stimulated Nck1 deficient cells. Furthermore, the F-actin/G-actin ratio was examined by Triton-X100 fractionation. Importantly, Nck1 knocked-out cells had a lower F/G actin ratio than control Jurkat T cells (Fig. 5b), indicating defective actin filament formation. To investigate impaired F-actin formation because of Nck1 depletion, wild-type *nck1* was introduced into Nck1-knocked Jurkat T cells (Fig. 1a). Re-constitution with wild-type *nck1* restored F-actin formation in these cells after stimulation (Fig. 5c). Altogether, Nck1 depletion and blocking of the Nck–CD3 interaction impaired actin polymerisation.

## Discussion

We studied the role of Nck in several aspects of lipid metabolism in T cells. Previously, we have shown that Nck1 is essential for TCR-mediated T cell activation and that two members of Nck family, Nck1 and Nck2, play non-redundant roles in TCR-triggered T cell stimulation [10]. Subsequently, the SH3.1 and SH2 domains of Nck have been found to be required for mediating Nck recruitment to CD3ε [13], which is essential for actin cytoskeleton rearrangement and promote TCR triggering [19]. Currently, the roles of Nck1 on lipid content and lipid metabolites have been mainly assessed. Nck1 deficiency was associated with increased lipid content in Jurkat T cells. In addition, inhibition of Nck–CD3 binding with the small molecule AX-024 caused an increase in the lipid profiles of Jurkat T cells upon stimulation. Importantly, the increased lipid content in Nck1 deficient cells upon TCR triggering was augmented by AX-024 treatment. AX-024 selectively interacts with SH3.1 of both Nck1 and Nck2 and SH3.1, both of which can be recruited to the CD3 PRS domain upon TCR engagement [20]. AX-024, Thus, inhibits the binding of both Nck1 and Nck2 to CD3ε. Nck1 and Nck2 play non-redundant roles in TCR-mediated T-cell activation, with Nck1 being the most important [11]. Although AX-024 induced an increase in lipid content in Nck1 knocked-out cells, the role of Nck2 could not be excluded. Therefore, the induced enhancement of lipid accumulation in Nck1-depleted cells by AX-024 might be due to its synergistic action with Nck2, which requires further investigation. Little is known about the role of Nck in lipid metabolism in T cells. However, in pancreatic β-cells, down-regulation of Nck1 is associated with increased activation of PERK (the PKR-like endoplasmic reticulum (ER) kinase) [21]. It has also been shown that silencing of Nck2 causes an increase in the levels of activated PERK and ATF4 (activating transcription factor 4) in 3T3-L1 adipocytes [22]. PERK is a component of the unfold protein response that plays a key role upstream of transcription factor ATF4 in fat storage and lipogenesis by inducing the expression of lipogenic genes in various cell types [23]. Increased activation of the PERK in Nck2 deficient cells can upregulate the lipogenic gene expression during adipocyte differentiation [22]. Interestingly, the SH2 domain of Nck1 and Nck2 has been found to mediate the interaction with phospho-PERK at Tyr-561 (Y561) in mouse insulinoma MIN6 cells and limit PERK activation [21]. In response to ER stress of pancreatic β-cell, Nck is dissociated from PERK and causes the dephosphorylation on the PERK pY561, consequently leading to PERK activation [21]. From these findings, we postulated that more lipid droplets in Nck1 deficient Jurkat T cells in this present study might be due to the increased activation of PERK and induced expression of lipogenic genes. To our surprise, blocking Nck-CD3 interaction by AX-024 enhanced lipid accumulation in wild-type and Nck1-depleted Jurkat T cells upon cell stimulation. However, the molecular mechanism underlying the role of Nck-CD3 interaction in lipid metabolism is not known.

In addition to the lipid content, Nck1 deficiency is related to the accumulation of several lipid metabolites. However, there are no studies which have defined the roles of these lipid metabolites in T cell activation. In other cell types, some lipid metabolites, such as methyl oleate, methyl stearate, and methyl linoleate, have antifungal activity [24]. Methyl palmitate inhibits phagocytosis in rat Kupffer cells [25]. Methyl palmitate promotes TLR4 signalling, resulting in increased COX-2 expression level [26], which then metabolises arachidonic acid to generate prostaglandin E_2_ [27].

In cancer cells, methyl elaidate can bind to Bax and an inhibitor of p53, making it a potential anticancer molecule [28]. Methyl linolenate and methyl linoleate have an effect on pigment inhibition activity. Thus, these lipid metabolites are good candidates for melanoma treatment [29]. Methyl homogamma linolenate is the essential omega-6 polyunsaturated fatty acid that has effects on anti-atherosclerotic [30], anti-inflammation as well as anti-cancer activities [31]. Moreover, methyl nervonate may inhibit DNA polymerase beta and HIV reverse transcriptase in a 3D structural study. Therefore, methyl nervonate may be a good inhibitor of HIV-1 infection [32]. In pancreatic β-cells, methyl palmitoleate promotes cell growth and protects against cell death [33]. However, the role of this lipid metabolite in T cells has not been investigated. Methyl cis-11-eicosenoate, or eicosenoic acid (cis-11), is formed by the elongation of oleic acid, which is then converted to docosenoic acid and nervonic acid. In the human monocyte cell line (THP-1), eicosenoic acid (cis-11) promotes IL-1β and IL-6 production, but inhibits the production of IL-10 [34]. Thus, decreased eicosenoic acid (cis-11) in Nck1 deficient cells might affect cytokine production by T cells.

Methyl hexanoate and hexanoic acid are both commonly found in the cytoplasm. In the HepG2 hepatoma cell line, hexanoic acid was found to regulate the PI3K/Akt/mTOR pathway, which is required for inducing lipid metabolism in this cell type [35]. Increasing levels of methyl hexanoate and methyl elaidate in AX-024 treated Nck1-depleted cells upon activation correlated with an increase in lipid content and lipid droplets. Thus, the increase in these lipid profiles observed in activated Nck1 knocked out cells in the presence of AX-024 might be caused by the upregulation of methyl hexanoate and methyl elaidate. In contrast, the high levels of these lipid profiles in resting Nck1 deficient cells might be caused by the 13 lipid metabolites mentioned above. In the current study, Nck1 depletion modulated lipid metabolites and consequently might affect Jurkat T cell biology, signalling, and functions. Therefore, further investigation is required to elucidate the mechanisms underlying the role of Nck1 in lipid metabolism in T cells.

Upon TCR triggering, Nck1 and WASP promote actin polymerization through the activation of the Arp2/3 complex [12, 20, 36–38]. We have shown that knockout of *nck1* impaired actin cytoskeleton rearrangement; this was also observed when the Nck–CD3 interaction was inhibited. This finding was consistent with a previous report which showed that a mutation of the CD3 PRS to block its association with Nck strongly inhibited actin polymerisation in mice [39]. Thus, recruitment of Nck to the TCR-CD3 complex is not only essential for TCR signalling but is also required for regulating actin rearrangement. This hypothesis is strongly supported by the finding that WASP is recruited to the TCR upon TCR triggering [40]. Since WASP is constitutively associated with Nck, inhibiting the Nck–CD3 association blocks the recruitment of WASP to the TCR, impairing actin polymerisation.

In this study, Nck1 was found to regulate actin polymerisation and lipid metabolism in Jurkat T cells. Both activities are regulators of cell structure, cell membrane, and organisation of the cytoplasmic component [41]. Typically, the size and volume of Jurkat T cells are 7–10 μm and 2,100–2,400 μm^3^, respectively [19, 42]. Knockout of Nck1 from Jurkat T cells altered their cell morphology. This is partly caused by the impairment of actin polymerisation in Nck1 deficiency Jurkat T cells, which leads to the loss of cell structure organisation. Furthermore, Nck1 depletion also affects various lipid metabolites, which are important for lipid raft formation [43]. Lipid rafts are lipid microdomains found in the external leaflet that support cell membrane flexibility and TCR clustering [15]. Moreover, upon TCR triggering, lipid rafts function as docking sites for signalling proteins such as the Src family of proteins, Lck [44]. Lipid raft disruption impairs Lck and ZAP70 phosphorylation, as well as the formation of LAT signalosomes in T cells [45]. Thus, the alteration of lipid metabolites by Nck1 depletion may affect lipid raft formation, which then impedes TCR signal transduction.

## Conclusions

In addition to the essential role of Nck1 in actin rearrangement, Nck1 regulates lipid metabolite level within T cells. Inhibiting the function of Nck1 both by gene knockout using CRISPR/Cas9 and by blocking the Nck–CD3 interaction using an inhibitor causes an increase in the lipid content in Jurkat T cells.

## Methods

### Cell culture

The human Jurkat T cell line (E6-1 clone) was obtained from the American Type Culture Collection (ATCC, Rockville, MD, USA). The Jurkat variant with a deficiency of Nck1 (N1KO) was generated using the CRISPR/Cas9 system, as previously described [46]. Cells were maintained in complete RPMI 1640 medium containing 10% foetal bovine serum (FBS), 1% penicillin–streptomycin, and 1% GlutaMAX (all from Gibco, MA, USA) at 37 °C in a humidified chamber with 5% CO_2_.

### Re-expression of wild-type Nck1 in Nck1-depleted Jurkat cells

Nck1 knocked-out Jurkat cells (N1KO) were transfected with pEBB plasmid containing human Flag-tagged wild-type Nck-1, which was kindly provided by Prof. Bruce J. Mayer (Connecticut University Health Center, Farmington, CT, USA), as previously described [13]. After transfection, cells were cultured in 10 mL complete medium (RPMI 1640 + 10% FBS + 1% Penicillin streptomycin + 1% GlutaMAX) and incubated at 37 °C, 5% CO_2_ for 72 h. Cell lysates were used to analyse Nck1 expression level by western blotting using antibodies against Nck1, and GAPDH was used as the loading control. The PVDF membrane was carefully cut according to the size of the studied proteins before probing the membranes with the desired specific antibodies. Band intensity was quantitatively identified using ImageJ software and presented as the normalised value of Nck1 relative to GAPDH. Uncropped blot images are available in the Supplementary Information.

### Cell treatment, cell cytotoxicity, and morphological observation

AX-024 is a small-molecule inhibitor of the Nck–CD3ε- interaction [47]. In this study, AX-024 was used to inhibit the binding of Nck to CD3ε in Jurkat T-cells. Jurkat and N1KO cells at a density of 2 × 10^5^ cells were starved in a starvation medium (RPMI 1640 + 10 mM HEPES) in the presence or absence of 10 nM AX-024 at 37 °C for 30 min. Next, anti-CD3ε antibody (clone OKT3) (Invitrogen, CA, USA) was added at a concentration of 5 μg/mL to stimulate the cells at 37 °C for 5 min. The cytotoxicity of AX-024 on Jurkat T cells was assessed using PrestoBlue™ Cell Viability Reagent (Thermo Fisher Scientific, MA, USA). The cell suspension was placed into the slide gap of the Tomodish at room temperature and observed under a holotomographic microscope (Tomocube, Daejeon, Republic of Korea) to analyse the morphological characteristics of the cells. Cell circularity was measured using ImageJ software and is presented as the circularity index (A.U.).

### CD3 expression in wild-type Jurkat T cells and N1KO cells

Wild-type Jurkat T cells and N1KO cells were assessed for CD3 expression by staining with anti-CD3ε antibody (PerCP) (Invitrogen) for 30 min at 4 °C. Isotype control cells were stained with anti-IgG2a, kappa antibody (PerCP) (ImmunoTools GmbH, Friesoythe, Germany). The stained cells were analysed using flow cytometry (FACSCalibur, Beckton Dickinson, NJ, USA).

### Lipid content and cell mass of cells under a holotomographic microscope

The 3D quantitative phase images of Jurkat T cells and N1KO cells based on the Fourier diffraction theorem [48] were generated using a holotomographic microscope model HT-2H (Tomocube) that applies optical diffraction tomography (ODT) using two UPLSAP 60X (NA 1.2) water-dipping lenses (Olympus, Tokyo, Japan). Holotomographic microscopy is a 3D quantitative phase imaging technology that provides label-free high-resolution images of living cells. This microscope measures the speed of light passing through the intracellular components (refractive index [RI]) and compares it with the RI database to identify the observed components [49]. The RI of each component depends on its mass and distribution [50]. Lipids have specific RI values that differ from other intracellular components. Thus, the 3D RI distribution of lipid content can be specifically quantified in label-free living cells [51].

In this process, 100 μL of the cell suspension was placed into the slide gap of Tomodish at room temperature. The cells were analysed for lipid content and cell mass using a holotomographic microscope. Full details of the optical configuration have been previously described [52, 53]. An Oil Red O staining kit (Sigma-Aldrich) was used to determine the lipid content in cells by visualisation under an inverted microscope (Nikon Eclipse Ti-U, Tokyo, Japan) [54].

### Actin polymerization in Jurkat T cell and N1KO cells

To visualise actin polymerisation in Jurkat T cells and N1KO cells, 2 × 10^5^ cells in 100 μL starvation medium were treated with 10 nM AX-024 or left untreated. Cells were incubated at 37 °C for 30 min, followed by stimulation with 5 μg/mL anti-CD3ε antibody (clone OKT3) at 37 °C for 5 min. The cells were then fixed with 4% paraformaldehyde (Sigma-Aldrich) and rendered permeable with 0.1% Triton X-100 (Amresco, NY, USA). After washing with 1 × PBS, samples were blocked with 1% bovine serum albumin (Capricorn, CA, USA) and washed with 1 × PBS. Cells were then stained with rhodamine-phalloidin-conjugated FITC (Abcam, MA, USA) at a dilution of 1:200 and incubated at room temperature for 20 min [46]. Finally, cells were seeded into a Tomodish at a total volume of approximately 100 μL of the total volume and observed for green fluorescence signals under the holotomographic microscope (Tomocube). ImageJ software was used to measure the value of integrated density, area of selected cell, and mean fluorescence of background before calculating the corrected total fluorescence (CTCF) intensity using the following formula: CTCF = integrated density – (area of selected cell × mean fluorescence of background readings).

### Measurement of Fibrous/Globular actin in cells by Triton X-100 fractionation

Jurkat T cells and N1KO cells were stimulated with anti-CD3ε OKT3 antibody (Invitrogen, CA, USA) with or without 10 nM AX-024. After stimulation, the cells were lysed with Triton X-extraction buffer containing 0.3% Triton X-100, 5 mM Tris (pH 7.4), 2 mM EGTA, 300 mM sucrose, 2 μM phalloidin, 1 mM PMSF, 10 μg/mL leupeptin, 20 μg/mL aprotinin, 1 mM sodium orthovanadate, and 50 mM NaF and incubated for 5 min on ice. The cell suspension was centrifuged at 600 × *g* for 5 min. The supernatant containing G-actin was then collected. The pellet was lysed with 30 μL lysis buffer and incubated for 5 min on ice. The cell suspension was centrifuged and the supernatant containing F-actin was collected. Equal volumes of each fraction were boiled in 4 × sample buffer at 95 °C for 15 min [55]. Supernatants were loaded onto 10% acrylamide gel and transferred onto a PVDF membrane (Merck Millipore, Billerica, MO, USA). The PVDF membrane was carefully cut according to the size of the proteins of interest before overnight incubation with an anti-actin antibody (rabbit monoclonal; Cell Signalling, dilution 1:1000). Blots were then incubated with a 1:5000 dilution of horseradish peroxidase (HRP)-conjugated secondary antibody (Merck Millipore) for 45 min at room temperature, developed using the Immobilon Forte Western HRP substrate detection reagent (Merck Millipore), and exposed to Imagequant LAS 4000 (GE Healthcare, IL, USA). Band intensity was quantitatively identified using ImageJ software and is presented as the normalised value of F-actin to G-actin. Uncropped blot images are available in the “Supplementary Information.

### Lipid metabolomics analysis in Jurkat T cells and N1KO cells

To study the change of lipid metabolites in treated Jurkat and N1KO cells, 1 × 10^6^ cells in 100 μL starvation medium were treated with 10 nM AX-024 and they were incubated for 30 min at 37 °C, followed by stimulation with 5 μg/mL anti-CD3ε antibody at 37 °C for 5 min. This protocol was adapted from Bligh and Dyer [56] and was based on a ratio of chloroform: methanol: water (2:2:1). The optimal cell volume was re-suspended in 50 μL of TNMg buffer (Tris, NaCl, and MgCl_2_) to allow cell fractionation. The volume of water was adjusted to maintain the solvent ratios. Then, 160 µL of GC–MS/MS-grade chloroform was added to the cell samples, followed by incubation on ice for 1 h. Subsequently, 320 μL of GC–MS/MS-grade methanol was added to the samples, agitated, and shaken at 600 rpm at room temperature for 30 min. Next, 110 μL of GC–MS/MS-grade water was added with agitation at 600 rpm at room temperature for 30 min. An additional 160 μL chloroform was added with agitation again for 10 min before centrifugation at 13,500 × *g* for 10 min at room temperature. Most of the lower organic phase was removed using a pipette and put in 3 mL furnace glass vials. An equal volume of the lower organic phase was replaced with chloroform to maintain the aqueous phase in the micro centrifuge tube. The samples were agitated at 600 rpm for 30 min and spun at 13,500 × *g* for 10 min at room temperature. For the blow down set up step, all vials were placed in a 40 °C dry heat block with lids off. The glass pipettes were held at the large end and fitted into a tube connected to nitrogen gas flow. Nitrogen gas was gently blown over the liquid until evaporation, without breaking the surface of the liquid. The blown time depended on the volume of solvent and flow rate, typically around 15 min/mL of methanol:chloroform. Dried lipid samples were stored at -80 °C until analysis. Subsequently, the samples were analysed by gas chromatography-quadrupole time-of-flight mass spectrometry (GC-QTOF, Agilent Technologies, USA) coupled to a PAL auto sample system (CTC Analytics AG, Switzerland).

### Statistical analysis

All statistical analyses of the numerical data were performed using Student’s *t*-test and one-way analysis of variance (ANOVA) using GraphPad Prism version 8.0.0 (GraphPad Software, CA, USA). The *p*-values less than 0.05 were considered statistically significant.

## Supplementary Information

**Additional file 1.**

**Additional file 2.**

**Additional file 3.** Standardization of lipid metabolites analyzed by GC-QTOF. Lipid metabolites were determined by GC-QTOF. The lipid metabolites were separated by CP-Sil 88 (high polarity) column, which can detect 37 lipid metabolites. The retention times of each standard metabolite are provided.

**Additional file 4.** The concentration of lipid metabolites in Jurkat and Nck-knocked out Jurkat cells. The concentration of lipid metabolites of triplicate independent samples was measured by GC-QTOF. Twenty-eight lipid metabolites were detected in these cells. Peak area of each component indicated lipid metabolites concentration.

## Data Availability

The data generated and/or analysed in the present study are available in the article. Data on the standardisation of lipid metabolites by GC-QTOF and raw data on lipid metabolites from the samples are included in the supplementary files.

## Abbreviations

Nck: Non-catalytic region of tyrosine kinase
Lck: Lymphocyte-specific protein tyrosine kinase
CD3: Cluster of differentiation 3
TCR: T cell receptor
CD28: Cluster of differentiation 28
CD3ε: Cluster of differentiation 3 epsilon
SH: Src homology
SLP-76: SH2-domain-containing leucocyte protein of 76 kDa
WASP: Wiskott–Aldrich Syndrome Protein
Arp2/3: Actin Related Protein 2/3 complex
PRS: Proline rich sequence
TSAd: T-cell specific adaptor protein
ODT: Optical diffraction tomography
RI: Refractive index
GC-QTOF: Gas-chromatography-quadrupole time of flight mass spectrometer
F-actin: Filamentous actin
G-actin: Globular actin
COX-2: Cyclooxygenase 2
TLR4: Toll-like receptor four
ZAP70: Zeta-chain-associated protein kinase 70
LAT: Linker for activation of T cells
N1KO: Nck1 knocked-out
OKT3: Anti-CD3 epsilon clone OKT3
EGTA: Egtazic acid
PMSF: Phenylmethylsulfonyl fluoride
NaF: Sodium Fluoride
TNMg: Compound of Tris, NaCl and MgCl_2_
GC–MS/MS: Gas Chromatography–Tandem Mass Spectrometry
ns: No statistically significant difference
