# Supplementary figures and images for: Lack of Nck1 protein and Nck-CD3 interaction caused the increment of lipid content in Jurkat T cells
Source: BMC Mol Cell Biol. 2022 Jul 28;23:36. doi: 10.1186/s12860-022-00436-3 (PMC9330638; doi:10.1186/s12860-022-00436-3)

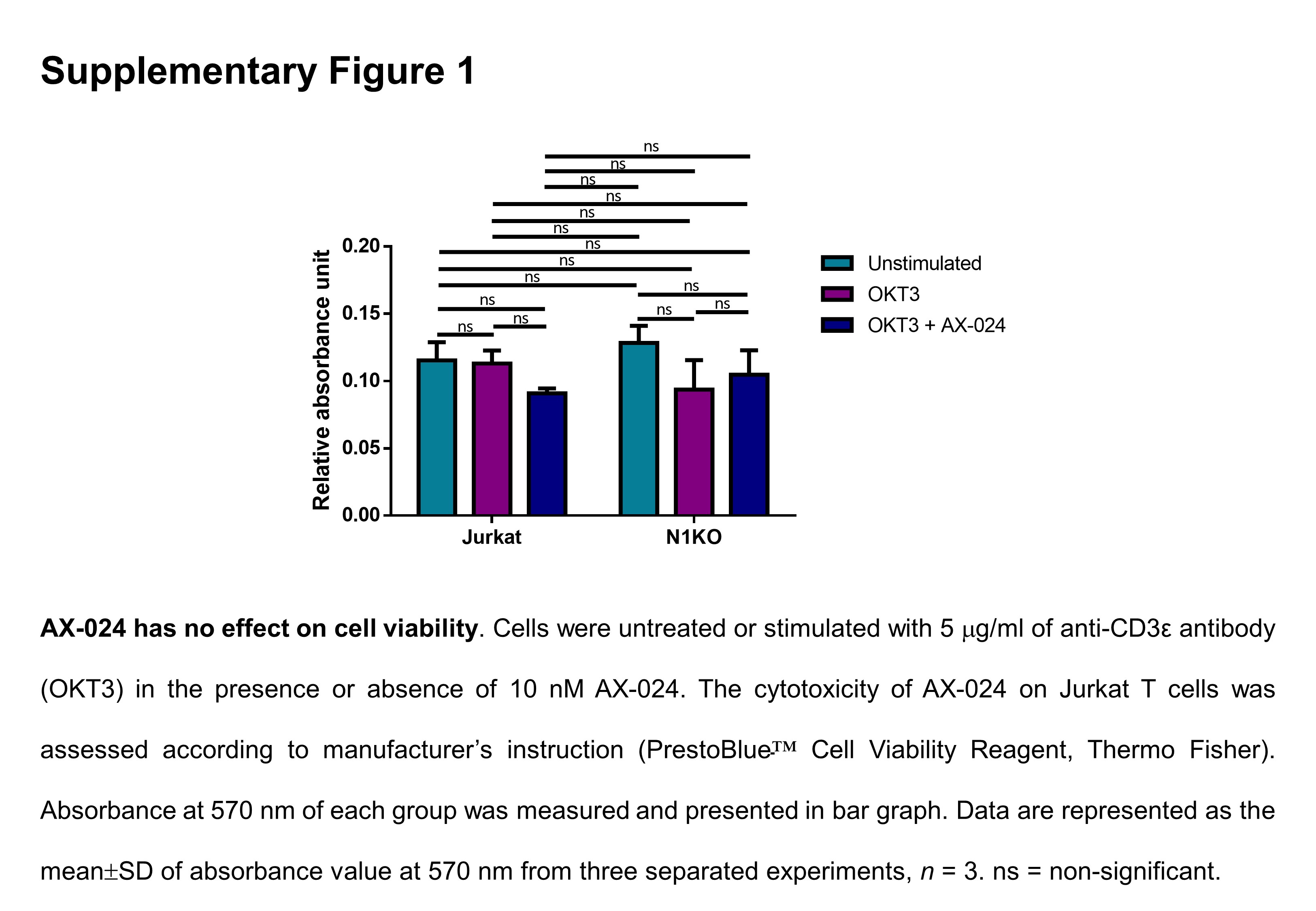

Supplement: Supplementary file 1 — Additional file 1. [file 12860_2022_436_MOESM1_ESM.jpg]
