# Supplementary material for: Lack of Nck1 protein and Nck-CD3 interaction caused the increment of lipid content in Jurkat T cells
Source: BMC Mol Cell Biol. 2022 Jul 28;23:36. doi: 10.1186/s12860-022-00436-3 (PMC9330638; doi:10.1186/s12860-022-00436-3)

**Supplementary File**

**Supplementary figure for Figure 1a.**

Original blotting images from five independent experiments were given, *n* = 5. The expression of Nck1 was analyzed by immunoblotting. Jurkat T cells were transfected with Nck1-specific CRISPR/Cas9 plasmid and Nck1-depleted Jurkat T cells were transfected with plasmid containing human Flag-tagged wild-type Nck-1. After transfection, the expression levels of Nck1, Nck2 and GAPDH were analyzed by immunoblotting. After being transferred, the PVDF membrane was carefully cut according to the size of the protein of interest in relation to the protein marker. The cut membranes were probed with the antibodies against Nck1, Nck2 and GAPDH. The images with short and long exposure times were provided. The squares indicated the studied proteins and the image with short exposure time was used to quantify the band intensity. Molecular weights in kDa were indicated in the left side of the images. M; marker lane.

For Figure 1a

Nck1 Nck2 and GAPDH – replicate 1


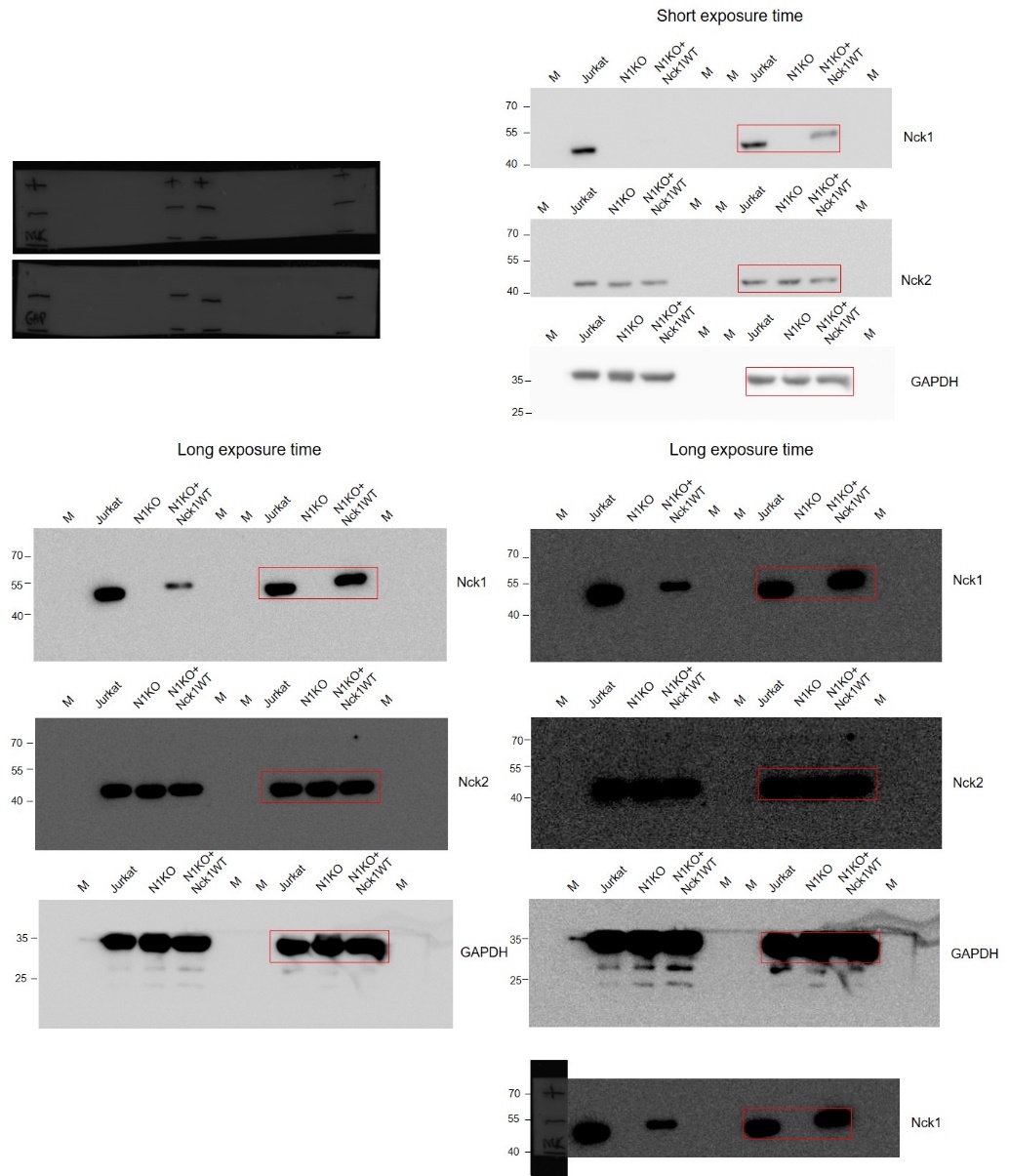


For Figure 1a

Nck1 Nck2 and GAPDH – replicate 2


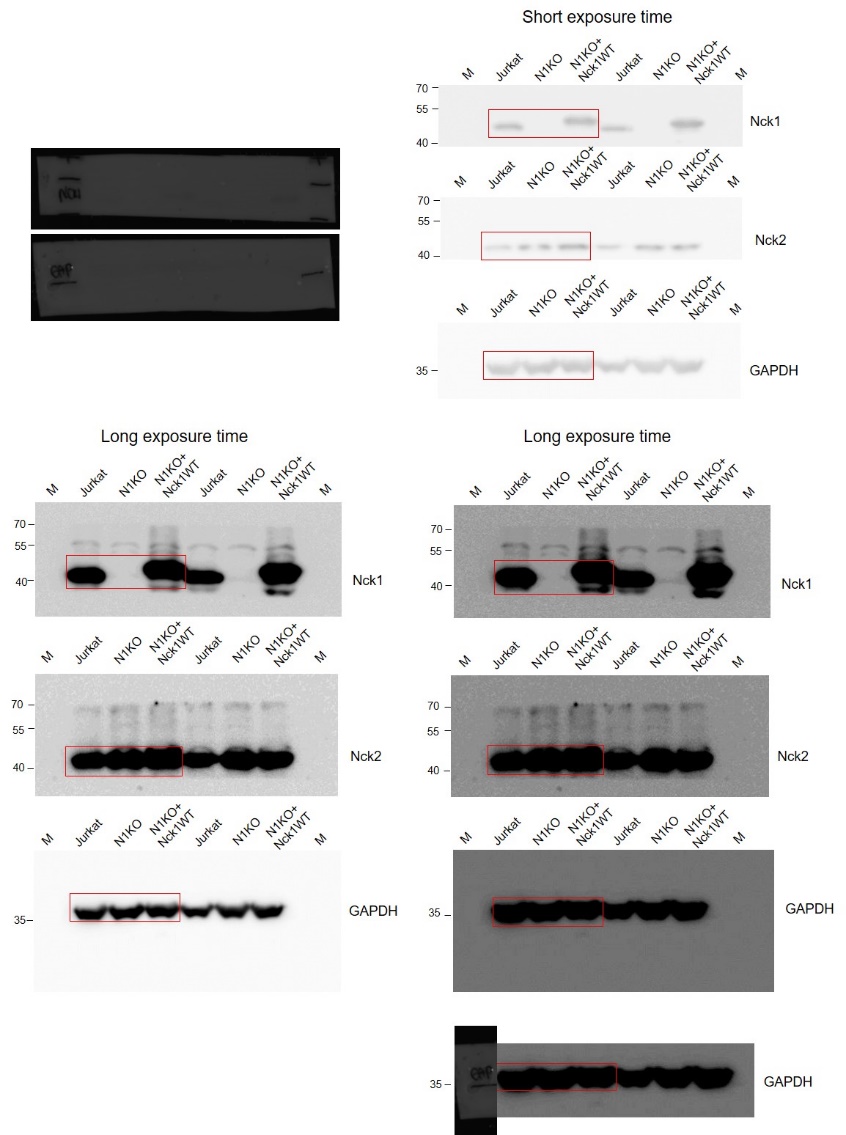


For Figure 1a

Nck1 Nck2 and GAPDH – replicate 3


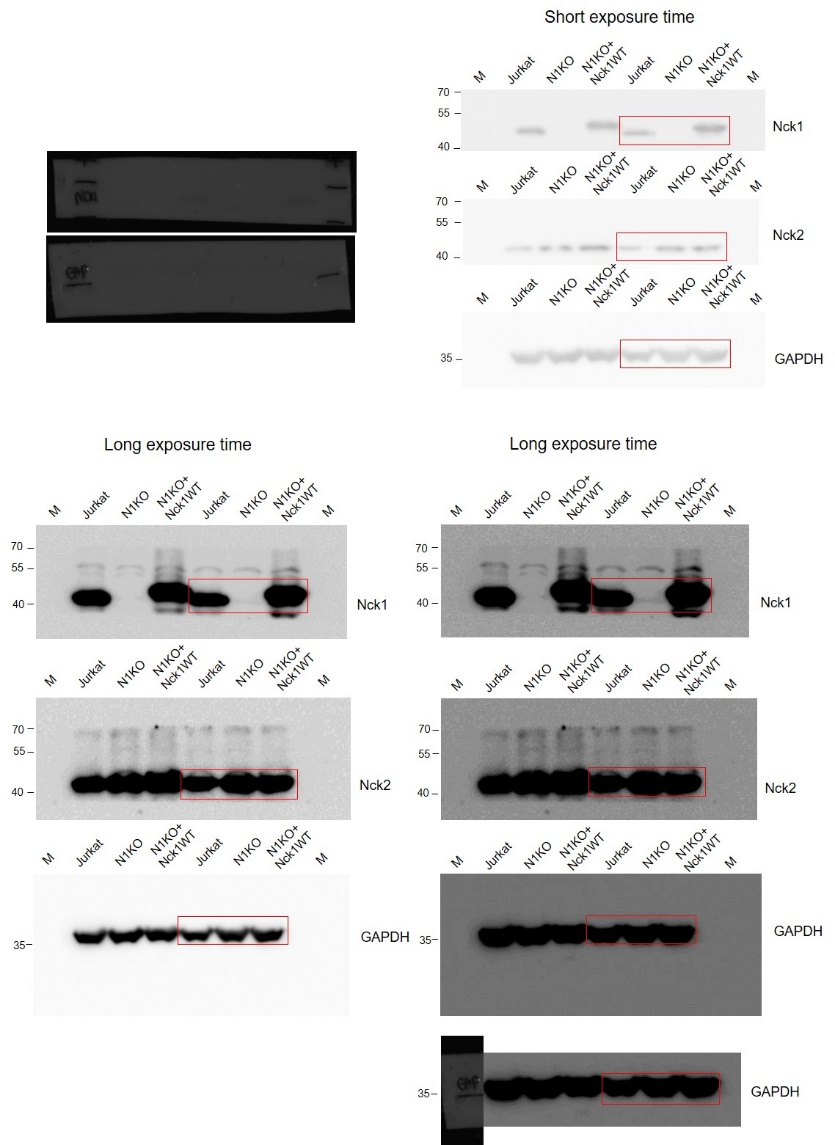


For Figure 1a

Nck1 Nck2 and GAPDH – replicate 4


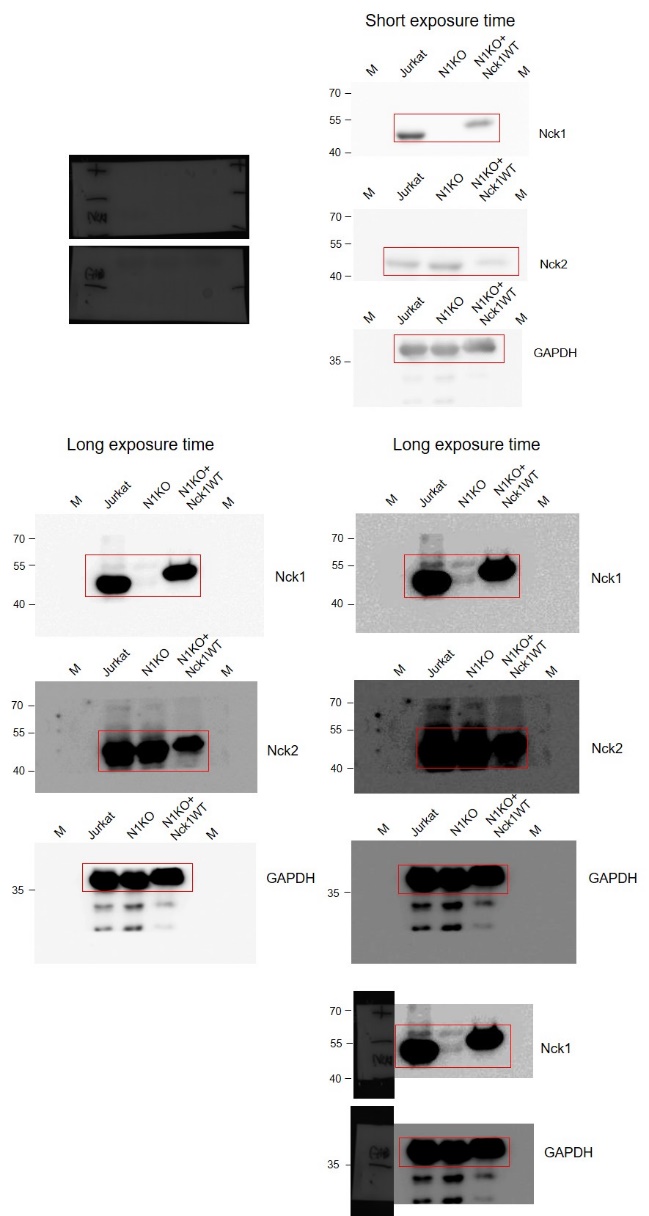


For Figure 1a

Nck1 Nck2 and GAPDH – replicate 5


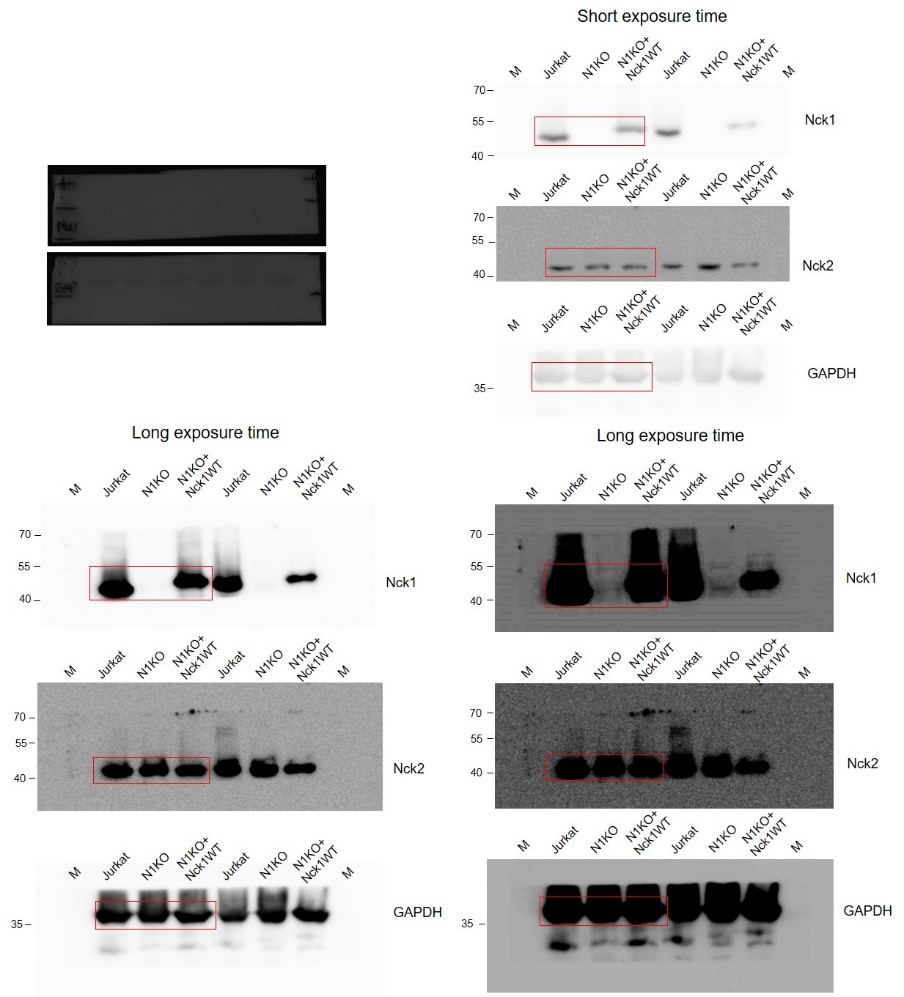


**Supplementary figure for Figure 5b.**

Original blotting images from three independent experiments were given, *n* = 3. F-actin/G-actin ratio in Nck1-depleted cells and Nck-CD3 interaction inhibition was analyzed by immunoblotting. Cells were untreated or stimulated with 5 μg/ml of anti-CD3ε antibody (OKT3) in the presence or absence of 10 nM AX-024. Then, fractions of G-actin and F-actin were prepared and subjected to immunoblot analysis. After being transferred, the PVDF membrane was carefully cut according to the size of the protein of interest in relation to the protein marker. The cut membranes were probed with the antibodies against actin. The blotting images with short and long exposure times were provided. The squares indicated the studied proteins and the image with short exposure time was used to quantify the band intensity. Molecular weights in kDa were indicated in the left side of the images. M; marker lane.

For Figure 5b

G-actin and F-actin – replicate 1


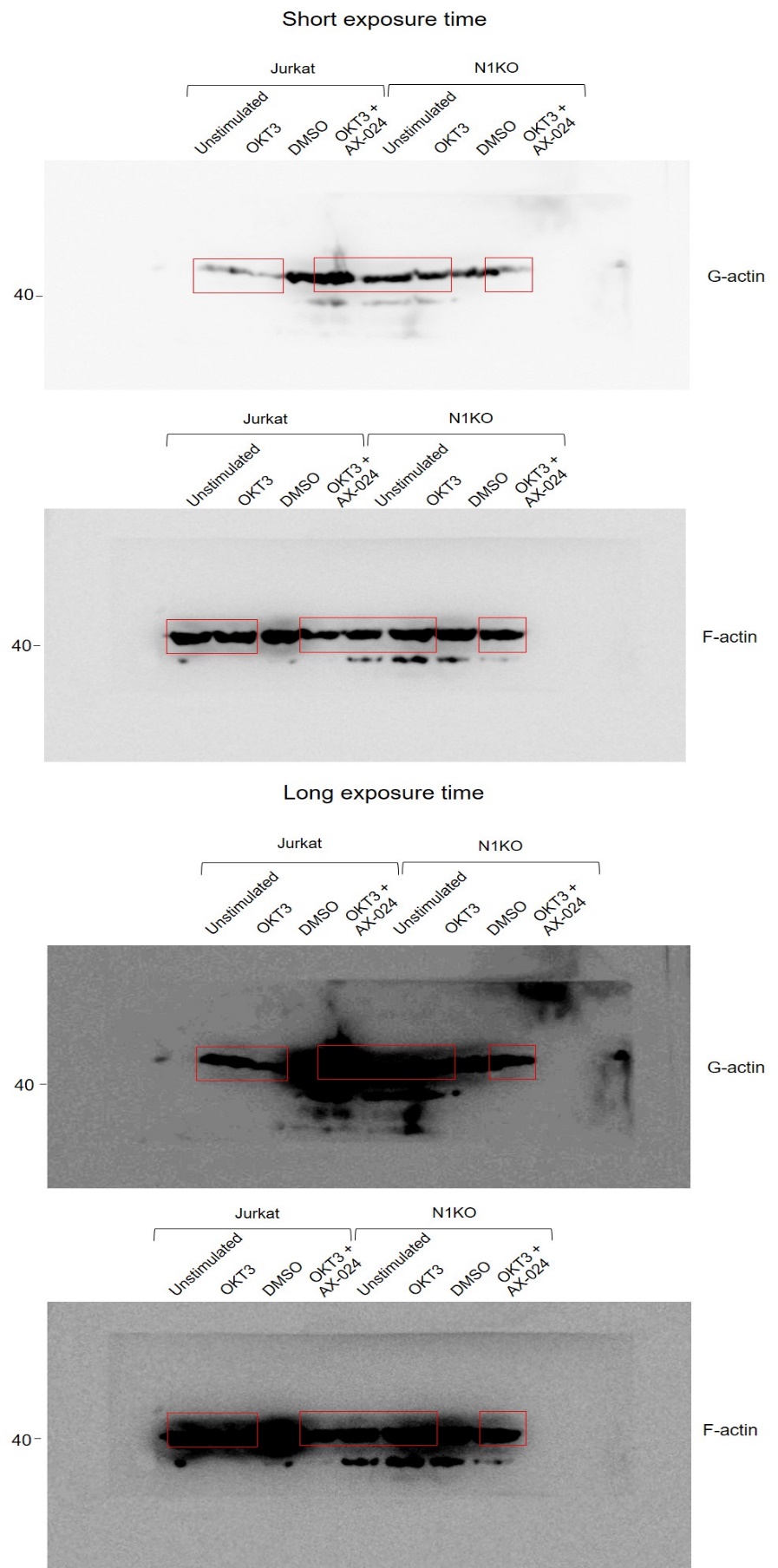


For Figure 5b

G-actin and F-actin – replicate 2


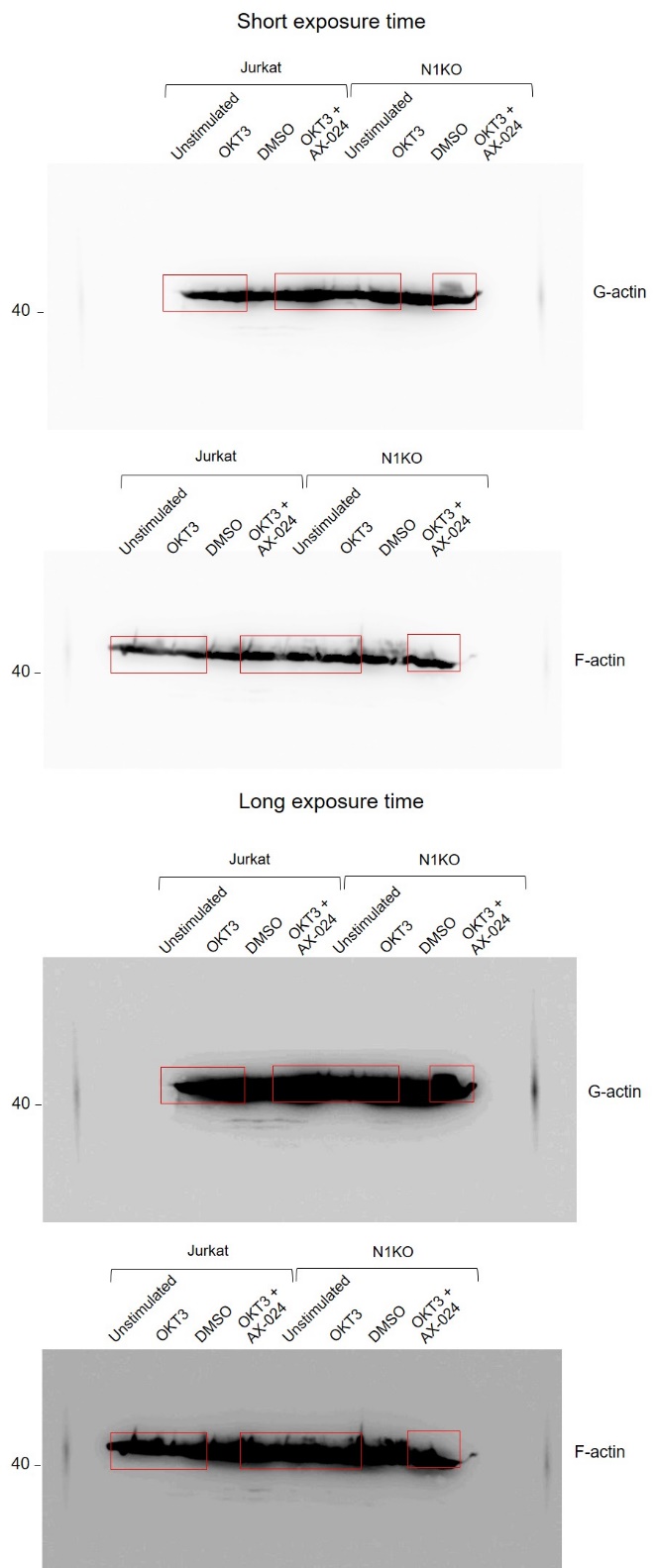


For Figure 5b

G-actin and F-actin – replicate 3


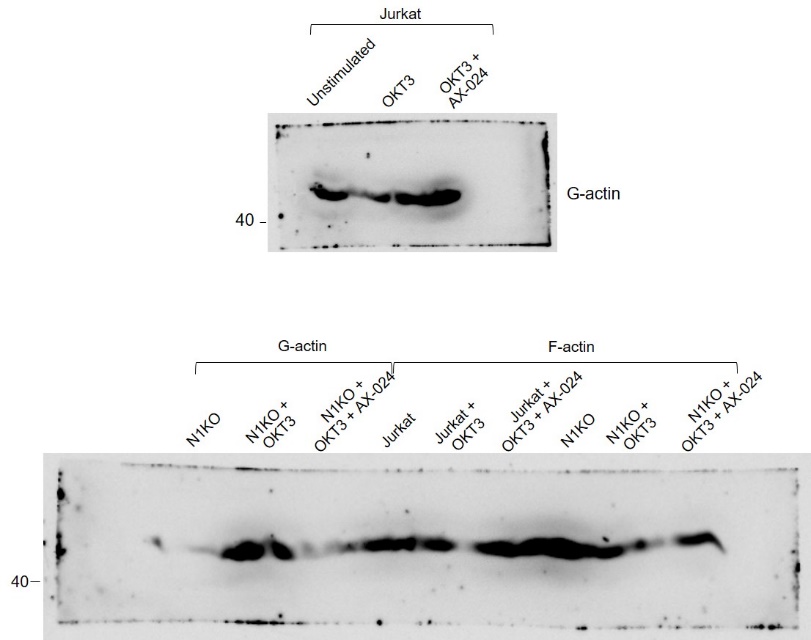

Supplement: Supplementary file 2 — Additional file 2. [file 12860_2022_436_MOESM2_ESM.docx]
