# Supplementary material for: Lack of Nck1 protein and Nck-CD3 interaction caused the increment of lipid content in Jurkat T cells
Source: BMC Mol Cell Biol. 2022 Jul 28;23:36. doi: 10.1186/s12860-022-00436-3 (PMC9330638; doi:10.1186/s12860-022-00436-3)

Standardization of Lipid metabolites analyzed by GC-QTOF			
Number	Chemical formula	Lipid metabolites	Retention time (min)
1	C4:0	Butanoic acid, methyl ester	9.224
2	C6:0	Hexanoic acid, methyl ester	9.898
3	C8:0	Octanoic acid, methyl ester	10.938
4	C10:0	Decanoic acid, methyl ester	12.324
5	C11:0	Undecanoic acid, methyl ester	13.111
6	C12:0	Dodecanoic acid, methyl ester	13.941
7	C13:0	Tridecanoic acid, methyl ester	14.805
8	C14:0	Methyl tetradecanoate	15.7
9	C14:1	Methyl myristoleate	16.565
10	C15:0	Pentadecanoic acid, methyl ester	16.645
11	C15:1 (cis-10)	methyl cis 10-pentadecenoate	17.578
12	C16:0	Hexadecanoic acid, methyl ester	17.653
13	C16:1 (cis-9)	9-Hexadecenoic acid, methyl ester, (Z)-	18.517
14	C17:0	Heptadecanoic acid, methyl ester	18.742
15	C17:1 (cis-10)	methyl cis-10-heptadecenoate	19.704
16	C18:0	Methyl stearate	19.944
17	C18:1 (trans-9)	9-Octadecenoic acid, methyl ester	20.585
18	C18:1 (cis-9)	9-Octadecenoic acid (Z)-, methyl ester	20.88
19	C18:2 (all-trans-9,12)	9,12-Octadecadienoic acid, methyl ester, (E,E)-	21.579
20	C18:2 (all-cis-9,12)	9,12-Octadecadienoic acid (Z,Z)-, methyl ester	22.253
21	C20:0	Eicosanoic acid, methyl ester	22.583
22	C18:3 (all-cis-cis -6,9,12)	6,9,12-Octadecatrienoic Acid Methyl Ester	23.308
23	C20:1 (cis-11)	cis-Methyl 11-eicosenoate	23.559
24	C18:3 (all-cis-cis -9,12, 15)	9,12,15-Octadecatrienoic acid, methyl ester, (Z,Z,Z)-	23.864
25	C21:0	Heneicosanoic acid, methyl ester	23.95
26	C20:2 (cis-11,14)	11,14-Eicosadienoic acid, methyl ester	25.0002
27	C22:0	Docosanoic acid, methyl ester	25.363
28	C20:3 (all-cis-8,11,14)	8,11,14-Eicosatrienoic acid methyl ester	26.157
29	C22:1 (cis-13)	13-Docosenoic acid, methyl ester, (Z)-	26.438
30	C20:3 (all-cis-11,14,17)	11,14,17-Eicosatrienoic acid, methyl ester	26.785
31	C23:0	Tricosanoic acid, methyl ester	26.899
32	C20:4n6	5,8,11,14-Eicosatetraenoic acid, methyl ester, (all-Z)-	27.1
33	C22:2 (all-cis-13,16)	Methyl Z,Z 13,16-docosadienoate	28.132
34	C24:0	Tetracosanoic acid, methyl ester	28.585
35	C20:5 (all -cis-5,8,11,14,17)	5,8,11,14,17-Eicosapentaenoic acid, methyl ester, (all-Z)-	29.227
36	C24:1 cis-15	15-Tetracosenoic acid, methyl ester, (Z)-	29.875
37	C22:6 (all-cis-4,7,10,13,16,19)	4,7,10,13,16,19-Docosahexaenoic acid methyl ester	34.949

Conditions

Technique:	GC-FID
Column:	Select CP-Sil 88 for FAME, 100 m x 0.25 mm df=0.2 µm (part number CP7489)
Oven:	80 °C, 4 °C/min to 220 °C, 5 min, 4 °C/min to 240 °C, 10 min
Carrier Gas:	Helium, constant flow 1.0 mL/min
Injection:	Splitflow 20 mL/min, Temperature = 250 °C
Injection Volume:	0.5 µL
Detection:	FID, 270 °C
Sample:	37 component FAME mix
Sample Solvent:	Dichloromethane
Sample Conc:	2-4% (w/w)

Peak Identification

1. C4:0
2. C6:0
3. C8:0
4. C10:0
5. C11:0
6. C12:0
7. C13:0
8. C14:0
9. C14:1
10. C15:0
11. C15:1
12. C16:0
13. C16:1
14. C17:0
15. C17:1
16. C18:0
17. C18:1 trans 9
18. C18:1 cis 9
19. C18:2, trans 9, 12
20. C18:2 cis 9,12
21. C20:0
22. C18:3 cis 6,9,12 gamma
23. C20:1 cis 11
24. C18:3 cis 9,12,15 alpha
25. C21:0
26. C20:2 cis 11,14
27. C22:0
28. C20:3 cis 8,11,14
29. C22:1 cis 13
30. C20:3 cis 11,14,17
31. C20:4 cis 5,8,11,14
32. C23:0
33. C22:2 cis 13,16
34. C24:0
35. C20:5 cis 5,8,11,14,17 EPA
36. C24:1 cis 15
37. C22:6 cis 4,7,10,13,16,19 DHA

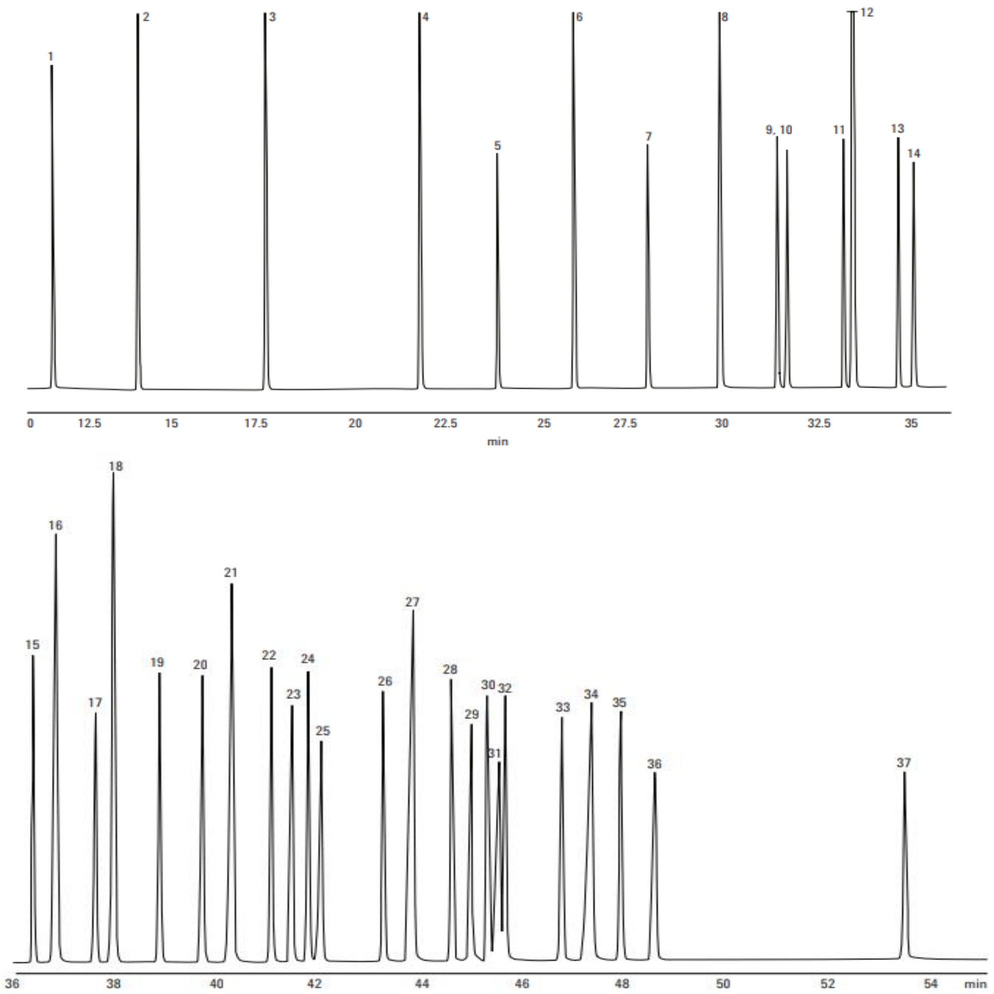

Supplement: Supplementary file 3 — Additional file 3. Standardization of lipid metabolites analyzed by GC-QTOF. Lipid metabolites were determined by GC-QTOF. The lipid metabolites were separated by CP-Sil 88 (high polarity) column, which can detect 37 lipid metabolites. The retention times of each standard metabolite are provided. [file 12860_2022_436_MOESM3_ESM.pdf]
