# Supplementary material for: Lack of Nck1 protein and Nck-CD3 interaction caused the increment of lipid content in Jurkat T cells
Source: BMC Mol Cell Biol. 2022 Jul 28;23:36. doi: 10.1186/s12860-022-00436-3 (PMC9330638; doi:10.1186/s12860-022-00436-3)
Supplement: Supplementary file 4 — Additional file 4. The concentration of lipid metabolites in Jurkat and Nck-knocked out Jurkat cells. The concentration of lipid metabolites of triplicate independent samples was measured by GC-QTOF. Twenty-eight lipid metabolites were detected in these cells. Peak area of each component indicated lipid metabolites concentration. [file 12860_2022_436_MOESM4_ESM.pdf]

Lipid metabolites concentration in Jurkat T cells analyzed by GC-QTOF (Peak area)									
Lipid metabolites	non-stimulated JK			OKT3 treated-JK			OKT3+AX024 treated-JK		
Hexanoic acid, methyl ester	0.331	0.370	0.312	0.439	0.413	0.426	0.364	0.348	0.312
Octanoic acid, methyl ester	0.491	0.569	0.620	0.724	0.561	0.677	0.526	0.588	0.518
Decanoic acid, methyl ester	0.408	1.608	0.743	0.512	1.167	1.609	1.513	0.504	1.126
Undecanoic acid, methyl ester	2.832	3.151	3.033	3.058	3.308	3.018	2.902	3.073	2.468
Dodecanoic acid, methyl ester	0.121	0.117	0.122	0.135	0.126	0.135	0.110	0.126	0.102
Tridecanoic acid, methyl ester	0.013	0.014	0.013	0.014	0.018	0.028	0.012	0.015	0.019
Methyl tetradecanoate	1.000	1.000	1.000	1.000	1.000	1.000	1.000	1.000	1.000
Pentadecanoic acid, methyl ester	0.243	0.221	0.220	0.233	0.238	0.215	0.248	0.227	0.226
methyl cis 10-pentadecenoate	18.448	21.111	19.783	20.589	20.324	18.555	20.013	19.946	18.623
Hexadecanoic acid, methyl ester	43.614	47.076	45.880	48.056	47.513	46.734	45.358	45.871	43.701
9-Hexadecenoic acid, methyl ester, (Z)-	2.766	2.934	2.984	2.717	2.706	2.479	2.782	2.704	2.847
Heptadecanoic acid, methyl ester	0.369	0.381	0.380	0.360	0.353	0.358	0.373	0.366	0.367
methyl cis-10-heptadecenoate	0.448	0.559	0.396	0.357	0.246	0.497	0.491	0.496	0.511
Methyl stearate	35.391	38.357	37.411	37.425	37.062	35.983	36.264	36.549	35.913
9-Octadecenoic acid, methyl ester	1.400	1.230	1.302	1.208	1.532	1.747	0.922	1.566	1.239
9-Octadecenoic acid (Z)-, methyl ester	37.956	40.757	40.990	37.012	36.568	35.481	38.960	37.690	39.950
9,12-Octadecadienoic acid (Z,Z)-, methyl ester	0.302	0.405	0.355	0.382	0.384	0.294	0.390	0.352	0.383
cis-Methyl 11-eicosenoate	0.167	0.178	0.170	0.180	0.177	0.169	0.174	0.171	0.168
9,12,15-Octadecatrienoic acid, methyl ester, (Z,Z,Z)-	1.442	1.702	1.743	1.483	1.615	1.500	1.673	1.484	1.816
Heneicosanoic acid, methyl ester	2.142	2.409	2.321	2.114	2.031	1.952	2.111	2.193	2.258
Docosanoic acid, methyl ester	1.721	1.755	2.050	1.402	1.923	1.731	1.659	1.704	1.966
8,11,14-Eicosatrienoic acid methyl ester	4.627	5.218	5.124	4.519	4.498	4.077	4.905	4.734	5.067
11,14,17-Eicosatrienoic acid, methyl ester	0.594	0.645	0.639	0.590	0.569	0.603	0.660	0.574	0.664
Tricosanoic acid, methyl ester	0.048	0.045	0.047	0.047	0.036	0.033	0.048	0.041	0.047
5,8,11,14-Eicosatetraenoic acid, methyl ester, (all-Z)-	0.209	0.449	0.205	0.183	0.284	0.223	0.261	0.370	0.282
Tetracosanoic acid, methyl ester	0.288	0.317	0.341	0.312	0.306	0.393	0.193	0.386	0.266
5,8,11,14,17-Eicosapentaenoic acid, methyl ester, (all-Z)-	0.030	0.042	0.034	0.049	0.047	0.043	0.043	0.042	0.046
15-Tetracosenoic acid, methyl ester, (Z)-	4.142	4.627	4.549	4.051	4.167	3.838	4.398	4.273	4.759

Lipid metabolites concentration in Nck1 knocked-out Jurkat T cells analyzed by GC-QTOF (Peak area)									
Lipid metabolites	non-stimulated N1KO			OKT3 treated-N1KO			OKT3+AX024 treated-N1KO		
Hexanoic acid, methyl ester	0.438	0.341	0.402	0.377	0.437	0.548	0.602	0.593	0.673
Octanoic acid, methyl ester	0.610	0.656	0.630	0.577	0.703	0.817	0.765	0.858	1.021
Decanoic acid, methyl ester	0.502	1.153	0.708	0.802	0.703	1.096	1.553	1.198	0.741
Undecanoic acid, methyl ester	3.811	3.195	3.008	3.354	0.703	4.527	4.420	5.119	5.146
Dodecanoic acid, methyl ester	0.131	0.136	0.157	0.171	0.703	0.125	0.334	0.218	0.265
Tridecanoic acid, methyl ester	0.027	0.013	0.016	0.015	0.703	0.034	0.036	0.037	0.024
Methyl tetradecanoate	1.000	1.000	1.000	1.000	0.703	1.000	1.000	1.000	1.000
Pentadecanoic acid, methyl ester	0.354	0.385	0.338	0.316	0.703	0.341	0.291	0.245	0.270
methyl cis 10-pentadecenoate	30.227	29.382	25.403	23.223	0.703	26.716	17.874	18.752	20.169
Hexadecanoic acid, methyl ester	55.746	54.768	49.938	44.694	0.703	56.384	42.404	46.447	49.371
9-Hexadecenoic acid, methyl ester, (Z)-	2.445	2.509	2.147	1.827	0.703	1.768	0.835	1.198	1.558
Heptadecanoic acid, methyl ester	0.725	0.753	0.637	0.534	0.703	0.648	0.338	0.379	0.408
methyl cis-10-heptadecenoate	0.297	0.480	0.301	0.292	0.703	0.474	0.921	0.181	0.128
Methyl stearate	44.323	45.166	39.052	34.259	0.703	42.209	27.021	29.805	31.927
9-Octadecenoic acid, methyl ester	2.069	1.904	1.690	1.471	0.703	1.723	2.397	2.622	2.571
9-Octadecenoic acid (Z)-, methyl ester	50.184	51.349	44.113	36.277	0.703	44.383	20.267	21.305	24.678
9,12-Octadecadienoic acid (Z,Z)-, methyl ester	1.914	2.042	1.781	1.471	0.703	1.808	0.941	1.001	1.113
cis-Methyl 11-eicosenoate	0.154	0.165	0.135	0.125	0.703	0.162	0.109	0.140	0.121
9,12,15-Octadecatrienoic acid, methyl ester, (Z,Z,Z)-	2.808	2.991	2.379	1.929	0.703	2.355	1.143	1.080	1.225
Heneicosanoic acid, methyl ester	2.401	2.319	2.157	1.703	0.703	2.082	0.921	0.925	1.162
Docosanoic acid, methyl ester	2.499	2.205	2.166	2.027	0.703	2.581	1.931	2.233	2.313
8,11,14-Eicosatrienoic acid methyl ester	10.248	10.271	9.014	6.953	0.703	8.402	3.477	3.668	4.326
11,14,17-Eicosatrienoic acid, methyl ester	0.761	1.279	1.113	0.858	0.703	1.086	0.463	0.514	0.616
Tricosanoic acid, methyl ester	0.040	0.045	0.037	0.027	0.703	0.036	0.024	0.021	0.031
5,8,11,14-Eicosatetraenoic acid, methyl ester, (all-Z)-	0.350	0.127	0.334	0.270	0.703	0.344	0.197	0.060	0.332
Tetracosanoic acid, methyl ester	0.436	0.415	0.335	0.369	0.703	0.456	0.048	0.845	0.555
5,8,11,14,17-Eicosapentaenoic acid, methyl ester, (all-Z)-	0.072	0.052	0.063	0.053	0.703	0.037	0.052	0.043	0.051
15-Tetracosenoic acid, methyl ester, (Z)-	8.007	8.485	7.205	5.518	0.703	6.836	2.970	3.140	3.586
